# Comparison of the Short-Term Outcomes after Postisometric Muscle Relaxation or Kinesio Taping Application for Normalization of the Upper Trapezius Muscle Tone and the Pain Relief: A Preliminary Study

**DOI:** 10.1155/2015/721938

**Published:** 2015-08-12

**Authors:** Kuba Ptaszkowski, Lucyna Slupska, Małgorzata Paprocka-Borowicz, Anna Kołcz-Trzęsicka, Kamil Zwierzchowski, Urszula Halska, Monika Przestrzelska, Dariusz Mucha, Joanna Rosińczuk

**Affiliations:** ^1^Department of Obstetrics, Faculty of Health Science, Wroclaw Medical University, K. Bartla 5, 51-618 Wroclaw, Poland; ^2^Department of Clinical Biomechanics and Physiotherapy in Motor System Disorders, Faculty of Health Science, Wroclaw Medical University, Grunwaldzka 2, 50-355 Wroclaw, Poland; ^3^Department of Physiotherapy, Faculty of Health Science, Wroclaw Medical University, Grunwaldzka 2, 50-355 Wroclaw, Poland; ^4^Department of Physiotherapy, Opole Medical School, Katowicka 68, 45-060 Opole, Poland; ^5^Institute of Biomedical Sciences, Academy School of Physical Education in Cracow, Aleja Jana Pawła II 78, 31-571 Kraków, Poland; ^6^Department of Nervous System Diseases, Faculty of Health Science, Wroclaw Medical University, K. Bartla 5, 51-618 Wroclaw, Poland

## Abstract

The main purpose of the study was to evaluate the resting bioelectrical activity of the upper trapezius muscle (the UT muscle) before and after one of the two interventions: postisometric muscle relaxation (PIR) and Kinesio Taping (KT). Moreover a comparison between group results was conducted. From the initial 61 volunteers, 52 were selected after exclusion criteria and were allocated randomly to 2 groups: PIR group and KT group. Outcome measures were assessed at baseline and completion of the intervention. The primary outcome measure was change in bioelectrical activity of UT muscle evaluated by surface electromyography (sEMG). Secondary outcomes included subjective assessment of pain using visual analogue scale (VAS). Significant differences were found only in KT group: the average resting bioelectrical activity decreased by 0.8 *μ*V (*p* = 0.0237) and the average VAS result reduced by 2.0 points (*p* = 0.0001). Greater decrease of VAS results was recorded in KT group compared to PIR group (*p* = 0.0010). Both PIR and KT intervention did not influence significantly the resting bioelectrical activity of UT muscle. KT application was better for pain relief in the studied sample compared with PIR intervention.

## 1. Introduction

Muscle energy techniques (MET) are a group of methods used in physiotherapy, osteopathy, and orthopedics as well as in sport training to treat all kinds of soft tissues dysfunctions [1–8]. One of the main objectives of MET is to reduce muscle tone of excessively strained muscles and also increase their extensibility [1–10]. From the point of view of terminology, the term MET includes postisometric relaxation technique (PIR). PIR is based on the active work of patient and therapist who exerts an optimal resistance. PIR is thought to induce change by reflex mechanisms. The result of this technique is a reduced tension within a muscle (or a group of muscles) and also increased muscle's tolerance to stretch, which is considered to be caused by stimulation of the Golgi tendon organs induced by isometric contraction [1–4, 10–15]. The methodology of PIR performance should take into account unchanging elements, such as finding the initial barrier of tissue resistance, giving a slight resistance by the therapist (isometric phase), relaxation, and finding a new end position (stretching). However, the realization of this technique may vary among the degree of isometric tension, the number of isometric and relax phases, the duration of the manual resistance, or the interval duration between consecutive series of PIR [1–6, 9, 16–29].

Another method which is often used to restore correct muscle tone is Kinesio Taping (KT) [30, 31]. KT is a therapeutic taping technique developed by Dr. Kenzo Kase (Japan, 1979). This method uses thin elastic tape which can be stretched up to 130–140% of its original length. Nowadays, KT is widely used in prevention of injuries, in rehabilitation of musculoskeletal disorders, and as support methods for the treatment of fascia, muscles, or joints dysfunctions [32–41] or even in lymphoedema [42–44]. Several studies have demonstrated a significant beneficial effect of KT, for example, increase of range of motion [33, 39], normalization of muscular function [30, 37], or diminishment of pain [31–33].

There have been no studies comparing PIR and KT. We undertook an evaluation of effectiveness of these two methods. The main purpose of the study was to evaluate the resting bioelectrical activity of the upper trapezius muscle (the UT muscle) before and after one of the two interventions: PIR and KT. Moreover, a comparison between group results was conducted. A secondary aim was to evaluate subjective perception of pain assessed by visual analogue scale (VAS).

## 2. Methods

### 2.1. Design and Approval

Prospective, controlled, randomized study comparing the PIR and KT effects. The study was approved by the Bioethics Committee of Opole Medical School (Poland) and all subjects provided written informed consent.

### 2.2. Subjects and Random Allocation of Patients

Volunteer participants were recruited from the Opole Medical School. Inclusion criteria were being 18 years of age or older, occurring pain and limited cervical range of motion during forward flexion and lateral flexion to the side opposite to the involved UT muscle, increased UT muscle tone (palpation assessment), the lack of skin allergies, and the consent to study participation. The exclusion criteria included any history of upper limb, back, or neck severe injury or medical intervention over the last 5 years (fracture, surgical intervention, and dislocation), peripheral or central nervous system neurological disease, pharmacological treatment at present, chronic headache, open wound, rash, infection, a pacemaker, or epilepsy.

From the initial 61 volunteers, 52 were selected after exclusion criteria and were allocated randomly to 2 groups: PIR group and KT group. The randomization was conducted using an electronic random number generator created by https://www.random.org/ that assigned each participant to either the PIR or KT group.

### 2.3. Outcomes

Outcome measures were assessed at baseline and completion of the intervention. The primary outcome measure was change in bioelectrical activity of UT muscle evaluated by surface electromyography (sEMG). Secondary outcomes included subjective assessment of pain during palpation assessment. The pain was recorded by the participant using a 10 cm VAS, where 0 represented no pain and 10 represented unbearable pain.

The intervention in PIR group (*n* = 26) consisted of three applications of postisometric relaxation technique on UT muscle within 24 hours (at regular intervals). In the KT group (*n* = 26) a 24-hour application of Kinesio Tex tape was used.

In PIR group, patients were in supine position with the neck side-bent away from the side being treated. Full side-bending was coupled with head supported in anteflexion and slightly rotated towards the side away from which the neck is bent. The arm on the side to be treated was lying alongside the trunk and was stabilized with one therapist's hand. The other therapist's hand was placed on the mastoid area. Postisometric relaxation technique was initiated with finding the position at the point at which the subject felt the very first sign of resistance (stretch feeling). Then, the subject was asked to take the stabilized shoulder towards the ear (using no more than 20% of available strength) against the resistance given by a physiotherapist. The isometric contraction phase was held for 7 seconds. The next step was a relaxation phase during which the subject was instructed to release his or her efforts. During relaxation, participants were asked to breathe in and out. While exhaling the therapist gently followed to the new resistance barrier. Each time this cycle was repeated three times.

The procedure in KT group involved a 24-hour “I” strip, tonus decreasing application with the use of Kinesio Tex Classic tape (Kinesio; 4 m/5 cm, Japan). Prior to tape application skin was cleaned from lotions, treated with alcohol, and shaved if needed. The length of the tape was measured from the acromion to the hairline in the elongated position. The base was affixed on acromion in neutral position. Then, the tape was applied on elongated UT by simply placing it on the muscle as it comes off of the paper backing, toward the hairline.

In the PIR group, measurements were taken before the first postisometric relaxation treatment and 10 minutes after the last intervention. In the KT group, measurements were made prior to the application and 10 minutes after the tape removal. All applications were performed by the same researcher (certified physiotherapist).

The electromyographic signal was registered by a dual-channel sEMG NeuroTrac ETS device (Verity Medical Ltd., United Kingdom) integrated with computer software for digital analysis and report creation. The device sensitivity is established at a level 0.1 *μ*V (4% accuracy; readings ± 0.3 mV at 200 Hz), with selectable bandpass filter (3 db bandwidth) and 50 Hz notch filter (33 dbs; 0.1% accuracy). This device is characterized by an amplitude range of 0.2–2000 *μ*V RMS continuous in the frequency band of 2–100 Hz and pulse width from 50 to 450 *μ*S for recording signals generated by muscles. The analogue signal recorded by the sEMG electrodes was amplified, filtered, and subsequently transformed into a digital signal. Mean values of muscle resting bioelectrical activity were given according to root mean square algorithm (RMS). The monopolar, self-adhesive reference electrode was placed on the seventh cervical vertebra. The electrodes were attached parallel to the muscle fiber orientation over the following muscles: at the UT muscle half way between the seventh cervical vertebra and the acromion (http://www.seniam.org/).

### 2.4. Statistical Analysis

Data were analyzed with the Statistica version 10 for Windows (Statsoft Inc., USA), and the results are presented as the mean ± SD. In order to analyze the changes in bioelectrical activity and VAS scale between pre- and postintervention results, the Wilcoxon matched-pairs test was used to examine the changes within each group. An independent and nonparametric Mann-Whitney *U* test was used for comparison among the two groups. A value of *p* < 0.05 was considered statistically significant.

## 3. Results

Sixty-one volunteers were screened for eligibility criteria. Fifty-two patients satisfied the criteria and were randomized into either the PIR group (*n* = 26; age: 20.4 ± 1.3) or KT group (*n* = 26; age: 20.6 ± 1.5) (Table 1). The reasons for ineligibility were previous history of upper limb, back, or neck severe injury (*n* = 5), not consenting to participation in the study (*n* = 2), and chronic headache (*n* = 2). There were no statistically significant differences between the PIR and KT group for any demographic measure (Table 2).

**Figure 1:**
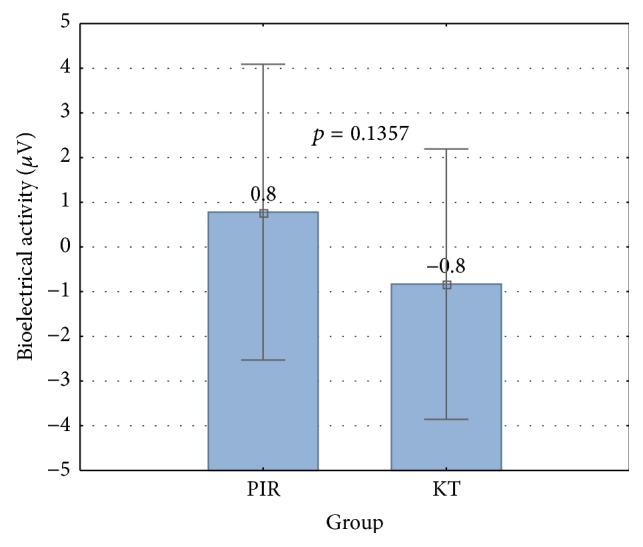
Comparison of bioelectrical activity results (post- minus preintervention results) between PIR and KT group (the Mann-Whitney *U* test).

Primary outcomes.  Figure 1 shows the comparison of changes of bioelectrical activity between PIR and KT groups. There was no significant difference of resting bioelectrical activity between the groups (*p* = 0.1357; Figure 1). The effects of PIR and KT on resting bioelectrical activity of UT muscle in each group are shown in Table 2. No significant differences were found between pre- and postintervention results in PIR group. The average resting bioelectrical activity increased by 0.8 *μ*V from preintervention to postintervention (*p* = 0.7971). Significant differences were found in KT group, and the average resting bioelectrical activity decreased by 0.8 *μ*V (*p* = 0.0237).

**Figure 2:**
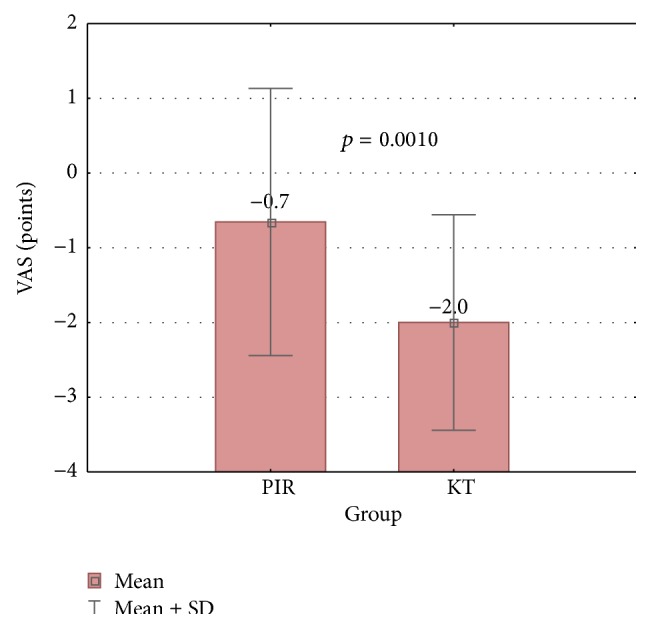
Comparison of VAS results (post- minus preintervention results) between PIR and KT group (the Mann-Whitney *U* test).

Secondary outcomes: Figure 2 shows the comparison of VAS results between PIR and KT groups. A statistically significant interaction was identified for VAS results. Greater decrease of VAS was recorded in KT group compared to PIR group (*p* = 0.0010; Figure 2). The effects of PIR and KT on VAS results are shown in Table 3. In PIR group, the average VAS result decreased by 0.7 points from preintervention to postintervention (*p* = 0.0654). Significant differences were found in KT group, and the average VAS result decreased by 2.0 points (*p* = 0.0001).

## 4. Discussion

The present research was designed to assess the short-term effects of two different therapeutic techniques on resting bioelectrical activity and pain relief within the UT muscle. The study found no difference in sEMG findings between groups; however, a difference in pain scores between groups was found. The obtained results show that PIR method did not change the sEMG activity and VAS score. However, KT application slightly decreased bioelectrical activity of the UT muscle as well as pain perception.

### 4.1. The Influence of PIR and KT on Bioelectrical Activity

In the intergroup analysis, there were no significant changes in sEMG in both groups and there are no studies which compare the PIR and KT methods. The results of the present study refer to the lack of significant change of resting bioelectrical activity after PIR. Only a limited number of studies have been reported evaluating the influence of MET on bioelectric signals recorded from muscles [4, 9, 11, 41]. The term “postisometric relaxation” is sometimes mistakenly equated with the contract-relax technique (CR) from the proprioceptive neuromuscular facilitation method (PNF), which results from the obvious analogy involving the use of isometric contraction, and the fact that the stretched muscle is contracted first and then relaxed. The essential difference is much greater force which the subject has to use to oppose the resistance given by the therapist in the CR technique. However, the mechanisms responsible for changes in muscle flexibility generated by PIR and CR remain questionable [1–7, 9, 16, 17, 20]. Some of the researchers [4, 9, 11, 41] using surface electromyographic measurement and analyzing the CR and PIR techniques have confirmed the reduction of bioelectrical activity of muscles treated with those stretching methods. Carter et al. studies [11], which were carried out on 24 healthy women, showed that hamstrings CR stretching decreased muscle tension in comparison to control group. The decrease in tonus, as explained by the author, may be due to desensitization of the muscle fibers within the muscle undergoing the stretch. Such results do not comply with Magnusson's findings [4, 5, 41, 45, 46]. In his studies, by assessing and comparing different alternatives of isomeric contraction of the hamstrings, he found that sEMG activity was unchanged after stretching procedures [4, 5, 41, 45, 46]. On the contrary, Ferber et al.'s [9] analysis suggested that both MET PIR and CR PNF techniques produce not only greater ROM but also greater bioelectrical activity within the stretched muscle.

Several studies have explored the impact of KT on bioelectrical muscle activity [30, 31, 35, 36, 47–53]. This study provides information about its positive effects on sEMG normalization. Similarly, Aguilar-Ferrándiz [31] reported enhanced gastrocnemius muscle activity after KT application in postmenopausal women with mild chronic venous insufficiency. In an assessor-blinded randomized crossover study conducted by Takasaki et al. [52], a significant effect of the KT intervention in the UT on sEMG amplitudes was also found. Researchers used three different interventions, tensioned taping, nontensioned taping, and no-taping control, and assessed muscle activity during a static typing task. In conclusion, authors suggested that both tensioned and nontensioned taping across the UT muscle reduce its activity. In the research conducted on healthy subjects Gómez-Soriano et al. [30], similar to Huang et al. [35] and Słupik et al. [51], did not report the effects of decreased muscle tone. However, these studies were performed in healthy individuals which may have minimized the expected therapeutic effect of KT.

### 4.2. The Influence of PIR and KT on Pain Relief

The effect of PIR on pain relief is also controversial. Although few studies confirmed analgesic effect of this technique, the results of this paper did not show the impact of PIR technique on pain relief. Wilson et al. [54] suggested that PIR reduces the pain sensitivity in patients in low-back pain. The subjects treated with MET showed a significantly higher improvement than those in the control group. Cassidy et al. [55] assessed the immediate effect of a single application of MET or manipulation in patients with neck pain. The MET group comparing to manipulation group did not show a significant decrease in pain. However, according to Franke et al. [1], studies on the efficiency of MET on pain in patients with nonspecific back pain provide low-quality evidence and further investigation is needed.

Frequent positive effects of pain management are reported in studies on KT. In this study KT application decreased the pain perception. It seems that KT applications are often used to reduce pain, especially in patients with musculoskeletal disorders [30, 34, 36–39, 47, 56–58]. A large number of studies have shown the positive effect of KT tape on low-back pain [37, 47–49]. Moreover, the usefulness of KT as an additional component of a physiotherapy program in patients with chronic nonspecific low-back pain was observed by Added et al. [50], which is partly supported by studies of Castro-Sánchez et al. [48]. Different findings were observed by Kachanathu et al. [47] In the comparison of physical therapy exercise program and KT application in nonspecific low-back pain, the authors did not notice the significant effect of KT.

## 5. Conclusion

Both PIR and KT intervention did not influence significantly the resting bioelectrical activity of UT muscle. KT application was better for pain relief in the studied sample compared with PIR intervention. Additionally, KT application was effective in reducing bioelectrical activity in intragroup analysis. Studies including large sample sizes and examining long-term effects (follow-up periods) are needed.

## Figures and Tables

**Table 1:** Characteristic of groups.

	Group		*p* value (^*∗*^Mann-Whitney *U* test; ^*∗∗*^Yates chi-square test)
	PIR	KT	
Number of patients	*n* = 26	*n* = 26	—

Age [year] Mean (SD) Range	20.4 (1.3) 19.0–25.0	20.6 (1.5) 19.0–25.0	*p* = 0.7281^*∗*^

Height [cm] Mean (SD) Range	172.8 (7.4) 157.0–184.0	169.1 (7.4) 158.0–188.0	*p* = 0.0570^*∗*^

Weight [kg] Mean (SD) Range	66.9 (12.2) 47.0–103.0	62.6 (11.0) 42.0–88.0	*p* = 0.1508^*∗*^

BMI [kg/m^2^] Mean (SD) Range	22.3 (3.1) 17.1–30.8	21.8 (2.8) 15.4–27.0	*p* = 0.6212^*∗*^

Sex	Female, 18 (69%) Male, 8 (31%)	Female, 20 (77%) Male, 6 (23%)	*p* = 0.5317^*∗∗*^

**Table 2:** Comparison between pre- and postintervention of resting bioelectrical activity results in each group.

Outcomes	Group	Measurement		*p* value (Wilcoxon's test)
		Before intervention	After intervention	
Resting bioelectrical activity (*µ*V) Mean (SD) Range	PIR	4.1 (1.3) 1.3–6.4	4.9 (2.7) 1.3–14.7	*p* = 0.7971
	KT	4.0 (2.3) 1.0–11.2	3.2 (2.2) 0.4–9.3	**p = 0.0347**

**Table 3:** Comparison between pre- and postintervention of VAS results in each group.

Outcomes	Group	Measurement		*p* value (Wilcoxon's test)
		Before intervention	After intervention	
Visual analogue scale (VAS) Mean (SD) Range	PIR	6.1 (1.8) 3.0–10.0	5.4 (1.8) 0.0–8.0	*p* = 0.0654
	KT	6.2 (1.4) 1.0–8.0	4.2 (1.4) 1.0–8.0	**p = 0.0001**
